# Factors associated with occupational calling among psychiatric nurses: a Bayesian network model analysis

**DOI:** 10.1038/s41598-026-44809-6

**Published:** 2026-03-18

**Authors:** Yu Ai, Qiaoling Liao, Xuemei Shen

**Affiliations:** 1 Department of Geriatrics II, The Third Hospital of Mianyang (Sichuan Mental Health Center), Mianyang, 621000 Sichuan China; 2 The Second Department of Severe Psychiatry, The Third Hospital of Mianyang (Sichuan Mental Health Center), Mianyang, 621000 Sichuan China; 3 General Psychiatry Department, The Third Hospital of Mianyang (Sichuan Mental Health Center), Mianyang, China

**Keywords:** Psychiatric nurses, Occupational calling, Job involvement, Professional identity, Bayesian network model, Health care, Health occupations, Medical research, Psychology, Psychology, Risk factors

## Abstract

To investigate the current status of occupational calling among psychiatric nurses, identify its correlated factors, and explore the probabilistic dependencies and network structure using a Bayesian network model. In June 2024, a convenience sample of 216 psychiatric nurses was recruited from a tertiary Grade A hospital in Mianyang City. Participants completed a questionnaire including demographic characteristics and scales for occupational calling, job involvement, professional identity, and work-family conflict. LASSO regression was used to screen key factors associated with occupational calling, and Bayesian network analysis was performed to explore the interrelationships among these factors. The mean score for occupational calling was 3.72 ± 0.65.Mean scores for job involvement, professional identity, and work-family conflict were 4.50 ± 1.36, 5.48 ± 0.74, and 2.61 ± 0.67, respectively. LASSO regression identified educational level (*β*=-0.19),nursing position(POST)(*β* = 0.08), professional title (*β* = 0.01), employment type (*β* = 0.06), professional identity (*β* = 0.20), work-family conflict (*β* = -0.19), and job involvement (*β* = 0.13) as factors associated with occupational calling, with professional identity showing the strongest association. The Bayesian network further revealed that POST, professional title, work-family conflict, and job involvement were directly associated with occupational calling, whereas educational level and employment type were indirectly linked to occupational calling through professional identity and job involvement, respectively. LASSO coefficient path analysis indicated that professional identity and job involvement maintained stronger and more stable associations across varying penalty levels, while demographic factors showed weaker and less stable associations. Occupational calling among psychiatric nurses is associated with a combination of demographic and psychosocial factors. LASSO coefficient path analysis demonstrated that psychosocial factors—particularly professional identity and job involvement—showed stronger and more stable associations with occupational calling than demographic characteristics whereas Work-family conflict exhibited a stable negative association throughout the regularization path. These findings provide preliminary evidence that strategies targeting the enhancement of professional identity, promotion of job involvement, and alleviation of work-family conflict may be associated with higher levels of occupational calling in this population.

## Introduction

The occupational calling of nursing staff, defined as their inherent sense of vocational mission and meaning, is closely linked to maintaining high-quality nursing services, ensuring patient safety, and stabilizing the nursing team. However, due to the particularity of their work, occupational calling among nurses is often reported at moderate to low levels, a pattern that may relate to the sustainable development of nursing teams^1^. Psychiatric nurses face highly specific workplace conditions: continuous exposure to psychiatric symptoms, coping with potential aggressive risks, and engagement in communication-intensive emotional labor. These cumulative factors may increase the risk of compassion fatigue and psychological trauma among psychiatric nurses, with both constructs showing negative associations with occupational calling^2^. Therefore, exploring the association patterns of their occupational calling is essential for understanding factors related to team retention and care quality. Extant research has demonstrated that occupational calling is shaped by the intricate interrelationships of individual attributes, organizational environment, and work-specific factors^1,3^. Nevertheless, most prior studies rely on cross-sectional surveys or single-dimensional mediation analysis, which limits the ability to unpack the complex interrelationship mechanisms among multiple factors.

 Bayesian networks, also known as belief networks, are graphical models that express probabilistic dependencies (without implying causality) among variables and are increasingly recognized as useful tools for visualizing complex data structures^4,5^. However, direct Bayesian network learning may yield unstable or overfitted structures when the number of potential correlated factors is large relative to the sample size. Therefore, integrating a variable selection procedure prior to network construction is critical to improve model stability and interpretability. LASSO regression is particularly suitable for this purpose, as it shrinks coefficients and selects a parsimonious set of predictors by penalizing the absolute values of regression coefficients, thereby helping reduce overfitting and multicollinearity^4^.

While LASSO identifies factors associated with occupational calling, it does not reveal the interrelationships among them. In contrast, Bayesian networks complement LASSO by modeling probabilistic dependencies and visualizing network structure through DAGs, enabling a more comprehensive understanding of how multiple factors are jointly associated with occupational calling^5^. This integrated approach—combining the strengths of LASSO for variable selection and Bayesian networks for elucidating complex interconnections—can provide a data-driven framework to inform psychiatric nursing management practices.

Therefore, this study aims to develop a Bayesian network model to investigate the factors associated with occupational calling among psychiatric nurses and their interconnections.

## Materials and methods

### Study design and participants

This was a cross-sectional study conducted among psychiatric nurses in a tertiary Grade A hospital in Mianyang City via convenience sampling in June 2024. A total of 226 questionnaires were collected, ten of which were discarded due to missing data on key variables, with 216 participants finally included in the study, corresponding to an effective response rate of 95.58%. The sample size of 216 was considered adequate for Bayesian network modeling, referencing prior studies in mental health nursing that recommend a minimum sample size of 10–30 participants per node^5,6^. The ratio of parameters (conditional probability table entries) to observations was 1:12.8, which is well below the 1:5 threshold associated with overfitting risk. The study protocol was approved by the Medical Ethics Committee of the Third People’s Hospital of Mianyang City (Approval No. 174, 2023). Written informed consent was obtained from all participants prior to the survey.

### Data collection

All data were collected through questionnaire survey. E-questionnaire was created through Questionnaire Star. Researchers pre-tested the electronic questionnaire and revised items for clarity and relevance. The e-questionnaires were sent to participants through WeChat group consisting of psychiatric nurses. All participants read and understood the study purpose, procedures, potential risks and benefits, and guaranteed confidentiality and voluntary participation before data collection. Each participant provided informed consent after they agreed to participate in this study. Time needed to complete the survey was 10 to 20 min. Researchers checked the completed questionnaires through data base of Questionnaire Star, questionnaires with obvious omissions or logic errors would be removed.

#### Measures

*Demographics* Demographic characteristics included gender, age, fertility status, POST, professional title, and work experience. POST was categorized as staff nurse, nursing team leader, and head nurse; professional titles were classified into junior title, intermediate title, and senior title.

*Occupational Calling* The scale included 12 items as a unidimensional instrument, specifically adapted for Chinese nursing professionals via revision and localization by Pei Yujing et al.^7^. It employed a 5-point Likert scoring system, with responses ranging from 1 (“strongly disagree”) to 5 (“strongly agree”) to indicate the level of occupational calling, where higher total scores reflected a stronger sense of occupational calling. In the present study, occupational calling was measured by this scale, and the instrument demonstrated good internal consistency, with a Cronbach’s α coefficient of 0.92.

*Job Involvement* The scale included 3 dimensions with a total of 17 items, specifically revised for use by Zhang Yiwen et al.^8^. It employed a 7-point Likert scoring system, with responses ranging from 1 (“never”) to 7 (“always”) to indicate the level of nurses’ job involvement, where higher total scores reflected a higher level of job involvement. In the present study, job involvement was measured by this scale, and the instrument demonstrated good internal consistency, with a Cronbach’s α coefficient of 0.95.

*Professional Identity* The Professional Identity Scale includes 5 dimensions and 21 items. Each item uses a 7-point Likert scale, with scores ranging from 1 (“strongly disagree”) to 7 (“strongly agree”)^9^. A higher score indicates a higher level of professional identity. The Cronbach’s α coefficient of this scale in this study was 0.89.

*Work-Family Conflict* The Work-Family Conflict Scale adopts a 5-point Likert scale (1 = “strongly disagree”, 5 = “strongly agree”)^10^, with higher scores reflecting greater work-family conflict.

#### Statistical methods

Data were downloaded from Questionnaire Star and formatted as R-compatible datasets. All statistical analyses were conducted using R 4.5.2. For descriptive and univariate analyses, *P* < 0.05 was considered statistically significant. Descriptive statistics were presented as mean ± standard deviation (SD), frequency, or constituent ratio, as appropriate. Independent samples t-tests and one-way analysis of variance (ANOVA) were employed to compare differences in key outcome variables across nurses with distinct demographic characteristics (e.g., gender, POST, professional title).

*LASSO Regression* LASSO regression was performed using the glmnet package in R for variable selection. The optimal penalty parameter *λ* was determined via 10-fold cross-validation, and the *λ*.1se criterion was used to select the final model to prioritizing parsimony. All candidate variables were included and coded as described in the Measures section.

*Bayesian Network Analysis* Bayesian network analysis was conducted using the bnlearn package in R for network structure learning, with visualization and conditional probability inference implemented via Netica 32.0. The network structure was learned using the Max-Min Hill-Climbing (MMHC) hybrid algorithm, with the Bayesian Information Criterion (BIC) as the scoring function. The conditional probability tables (CPTs) were fitted by maximum likelihood estimation—consistent with the the fitting method of the bnlearn package—to ensure result uniformity.

For data preprocessing, continuous variables… were discretized into three ordinal levels (low, medium, high) via quantile-based equal-frequency binning, with cut-off points set at the 33.3rd and 66.7th percentiles. Categorical variables (education, POST, professional title, employment type) were included as originally classified, without additional discretization or recoding.

To assess the robustness of the learned network structure, bootstrap resampling with 200 replicates was performed using the Hill-Climbing (HC) algorithm, with BIC as the scoring function. The strength S(e) of each directed edge was calculated as its frequency of occurrence across the bootstrap samples, and edges with strength exceeding a predefined threshold (0.50) were considered stable.

In this study, the term “probability” refers to the conditional probabilities estimated by the Bayesian network model based on parent node states, does not imply causality.

## Results

### Demographic

Demographic characteristics of participants and scores of key outcome variables across different groups are presented in Table 1. The mean score of occupational calling was (3.72 ± 0.65), the professional identity was (5.48 ± 0.74), the job involvement was (4.50 ± 1.36), and the work-family conflict was (2.61 ± 0.67).Univariate analyses showed statistically significant differences in occupational calling scores across age groups, educational levels, positions, and night shift frequencies (*P* < 0.05); statistically significant differences in professional identity scores were observed among nurses with different fertility statuses, education, and POST (all *P* < 0.05); a statistically significant difference in job involvement scores existed among nurses with varying POST (*P* < 0.05); and statistically significant differences in work-family conflict scores were found among nurses with different fertility statuses, education, POST, and employment type (all *P* < 0.05).

**Table 1:** Demographic characteristics of Occupational Calling, Professional Identity, Job Involvement, and Work-Family Conflict Among Psychiatric Nurses in a Tertiary Grade A Hospital.

Characteristics	Number(*n* = 216, %)	Occupational calling	Professional identity	Job involvement	Work-family conflict
		Mean (SD)			
Age					
36–45 years old	70 (32.4%)	3.63 ± 0.68	5.53 ± 0.8	4.48 ± 1.46	2.46 ± 0.71
25–35 years old	108 (50%)	3.71 ± 0.65	5.44 ± 0.72	4.45 ± 1.35	2.62 ± 0.64
> 45 years old	21 (9.8%)	4.07 ± 0.58	5.58 ± 0.57	4.69 ± 1.25	2.98 ± 0.73
<25 years old	17 (7.8%)	3.76 ± 0.58	5.44 ± 0.84	4.63 ± 1.29	2.75 ± 0.44
*F* -value		8.103	0.786	1.02	7.027
*P* -value		0.0439	0.8529	0.7965	0.0711
Gender					
Male	25 (11.6)	3.59 ± 0.74	5.56 ± 0.75	4.43 ± 1.32	3.1 ± 0.51
Female	191 (88.4)	3.74 ± 0.65	5.47 ± 0.74	4.51 ± 1.37	2.55 ± 0.66
*T* -*value*		−0.812	0.4172	−0.812	0.4172
*P -v*alue		0.342	0.7335	0.342	0.7335
Fertility status					
1 children	101 (46.8)	3.81 ± 0.63	5.6 ± 0.76	4.55 ± 1.44	2.57 ± 0.75
2 children	53 (24.5)	3.63 ± 0.8	5.4 ± 0.73	4.73 ± 1.41	2.48 ± 0.68
No children	62 (28.7)	3.66 ± 0.54	5.37 ± 0.71	4.22 ± 1.15	2.79 ± 0.47
*F* -value		4.514	6.02	3.42	6.968
*P* -value		0.1046	0.0493	0.1808	0.0307
Education					
Associate degree	65 (30)	3.87 ± 0.6	5.37 ± 0.81	4.62 ± 1.36	2.72 ± 0.74
Bachelor’s degree	144 (66.7)	3.69 ± 0.67	5.57 ± 0.69	4.49 ± 1.37	2.55 ± 0.63
Master’s degree or above	7 (3.3)	3.18 ± 0.57	4.85 ± 0.68	3.5 ± 0.96	2.97 ± 0.64
*F* -value		6.462	7.689	4.452	6.882
*P* -value		0.0395	0.0214	0.108	0.032
POST					
Nurse	181 (83.8)	3.67 ± 0.68	5.42 ± 0.77	4.39 ± 1.35	2.66 ± 0.64
Nursing Team Leader	27 (12.5)	4.05 ± 0.46	5.95 ± 0.46	5.42 ± 1.21	2.2 ± 0.7
Head nurse	8 (3.7)	3.83 ± 0.13	5.42 ± 0.15	3.9 ± 0.78	3.03 ± 0.69
*F* -value		8.18	14.158	14.585	14.99
*P* -value		0.0167	0.0008	0.0007	0.0006
Professional title					
Junior professional title	81 (37.5)	3.59 ± 0.7	5.44 ± 0.73	4.26 ± 1.37	2.58 ± 0.62
Intermediate professional title	109 (50.5)	3.76 ± 0.64	5.49 ± 0.79	4.64 ± 1.39	2.56 ± 0.65
Senior professional title	26 (12)	3.99 ± 0.53	5.6 ± 0.58	4.64 ± 1.14	2.91 ± 0.85
*F* -value		4.0421	0.4436	1.9659	2.9465
*P* -value		0.0189	0.6423	0.1426	0.0547
Employment type					
Establishment staff	34 (15.7)	3.75 ± 0.64	5.5 ± 0.64	4.2 ± 1.18	2.85 ± 0.78
Contract based staff	182 (84.3)	3.72 ± 0.66	5.48 ± 0.76	4.56 ± 1.39	2.57 ± 0.64
*F* -value		0.0476	0.0085	1.9311	5.146
*P* -value		0.8275	0.9265	0.1661	0.0243
Work experience					
0~< 6 years	63 (29.6)	3.63 ± 0.58	5.27 ± 0.71	4.14 ± 1.27	2.66 ± 0.59
6~< 11 years	45 (20.8)	3.69 ± 0.79	5.47 ± 0.68	4.55 ± 1.48	2.44 ± 0.76
≥ 11years	105 (48.6)	3.77 ± 0.67	5.57 ± 0.73	4.51 ± 1.37	2.6 ± 0.7
*F* -value		0.4649	1.7536	0.7847	1.0864
*P* -value		0.629	0.1762	0.4579	0.3397
Night shift					
0	31 (14.4)	3.98 ± 0.47	5.45 ± 0.62	4.64 ± 1.08	2.73 ± 0.49
1–4	48 (22.2)	3.52 ± 0.8	5.41 ± 0.82	4.49 ± 1.43	2.58 ± 0.87
5–10	107 (49.5)	3.7 ± 0.63	5.44 ± 0.74	4.4 ± 1.42	2.61 ± 0.6
≥ 11	30 (13.9)	3.89 ± 0.55	5.8 ± 0.68	4.71 ± 1.35	2.57 ± 0.74
*F* -value		9.262	5.314	1.444	1.387
*P* -value		0.026	0.1502	0.6953	0.7086

### Occupational calling status LASSO regression analysis

Considering that LASSO regression can reveal the interactions and relative importance among multiple variables, all factors were included as input variables in the model. A total of 12 variables were assigned as independent variables, including age, gender, fertility status, education, work experience, POST, professional title, employment type, night shifts, job involvement score, professional identity score, and Work-family conflict score; detailed assignment criteria are presented in Table 2.

**Table 2:** Assignment of independent variables.

Variables	Assignment rules	Variables	Assignment rules
Age	Substitute the original value	Work experience	Substitute the original value
Gender	Male = 0;female = 1	Night shift	Substitute the original value
Education	College Below = 0; Bachelor Above = 1	Occupational calling	Substitute the original value
POST	Nurse = 0;Nursing Team leader = 1;Head nurse = 2	Professional identity	Substitute the original value
Professional title	Junior professional title = 0;Intermediate professional title = 1; Senior professional title = 2	Work-family conflict	Substitute the original value
Employment type	Contract based staff = 0;Establishment staff = 1	Job involvement	Substitute the original value
Fertility status	No children = 0;Have children = 1		

① Continuous variables (age, work experience, monthly night shifts, occupational calling, professional identity, work–family conflict, job involvement) were retained as original values. The Shapiro–Wilk test confirmed that these variables followed a normal distribution (all *P* > 0.05), which helped to preserve complete data information and avoid information loss caused by discretization. ② Due to the small sample size of nurses with a master’s degree or above (*n* = 7, 3.3%), education was dichotomized into “college above or below (0)” and “bachelor’s degree or above (1)”. Preliminary analysis using three educational categories yielded unstable parameter estimates; thus, dichotomization was employed to ensure model stability. ③ The coding for POST and professional title was nominal (not ordinal implications) and these variables were treated as categorical variables in all analyses. The coding order does not affect the inference of variable associations.

**Fig. 1:**
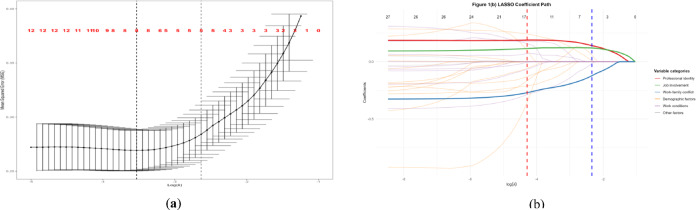
LASSO regression results. (**a**) Cross-validation Error for LASSO regression. (**b**) LASSO coefficient path plot. In both panels, red dashed line = *λ*.min, blue dashed line = *λ*.1se. In panel (b), colored lines represent coefficient trajectories: red is professional identity, green is job involvement, blue is work-family conflict, orange is demographic factors (age, gender, education, POST, professional title, employment type), purple is work conditions (work experience, night shifts, monthly income), gray is other variables. The upper x‑axis shows the number of non‑zero coefficients at each *λ*.

LASSO regression with 10-fold cross-validation was performed with occupational calling as the dependent variable. Figure 1(a) shows the cross-validation process for *λ* selection, with the vertical dashed line indicating the optimal *λ*.1se value (0.002148) selected by the one-standard-error rule. This parameter achieved a balance between parsimony and predictive stability, corresponding to a mean squared error (MSE) of 0.3785 (SD = 0.0521). The coefficient paths for all predictors across different *λ* values are illustrated in Fig. 1(b). Each colored line represents the trajectory of a variable’s coefficient as the penalty parameter varies, and the upper x-axis shows the number of non-zero coefficients retained in the model. Professional identity and job involvement maintained the largest coefficients throughout the regularization path, indicating their dominant associations with occupational calling. Based on the *λ*.1se criterion, seven predictors with non-zero coefficients were retained as potential correlated factors (Table 3): educational level (*β*= −0.19), POST (*β* = 0.08), professional title (*β* = 0.01), employment type (*β* = 0.06), professional identity (*β* = 0.20), work-family conflict (*β*= −0.19), and job involvement (*β* = 0.13).

**Table 3:** Variables selected by LASSO regression with 10-fold cross-validation.

Variables	LASSO regression beta coefficient (β)
Education	−0.19
POST	0.08
Professional title	0.01
Employment type	0.06
Professional identity	0.20
Work-family conflict	−0.19
Job involvement	0.13

① This table presents the core variables screened by LASSO regression; the absolute value of the coefficient reflects the relative strength of the association with occupational calling.② The optimal penalty parameter was determined by 10-fold cross-validation, with the model selected at*λ*.1se = 0.002148; the corresponding 10-fold cross-validation mean square error (MSE) was 0.3785 (SD = 0.0521), confirming the model’s stability and parsimony. ③ Education was dichotomized due to the small sample size of nurses with master’s degrees or above (*n*=7, 3.3%) to ensure stable parameter estimation.

### Bayesian network model for the occupational calling of psychiatric nurses

**Fig. 2:**
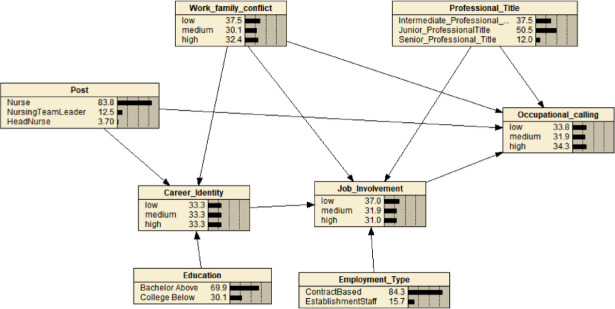
LASSO feature selection combined with Bayesian network probability inference model diagram.

Based on the 7 variables screened by LASSO regression, this study further constructed a Bayesian network model between the level of occupational calling and its related factors using the Max-Min Hill-Climbing (MMHC) algorithm, as shown in Fig. 2. The model consisted of 8 nodes and 10 directed edges. Bootstrap stability analysis (Supplementary Table 4) confirmed the robustness of the core network structure, with professional identity demonstrating the highest stability (S(e) > 0.80)for all connected edges). Job involvement (S(e) = 1.00), work-family conflict (S(e) = 0.965), professional identity (S(e) = 0.835–1.00), POST, and professional title were directly connected to occupational calling as parent nodes. Educational level and employment type were indirectly associated with occupational calling through job involvement and professional identity. Specifically, educational level was linked to occupational calling via professional identity (S(e) = 0.835), and employment type was linked via job involvement (S(e) = 0.835). This suggests that job involvement and professional identity constitute an indirect pathway connecting educational level and employment type with occupational calling.

### Structural stability of the Bayesian network

To assess the robustness of the learned network structure, we performed bootstrap resampling with 200 replicates using the Hill-Climbing (HC) algorithm with BIC as the scoring function. The strength S(e) of each directed edge, defined as its frequency of occurrence across the bootstrap samples, was calculated. Following common practice, edges with S(e) > 0.50 were considered stable.

The bootstrap analysis revealed that 6 edges exhibited high stability (S(e) > 0.50). The strongest connections were observed between job involvement and professional identity (S(e) = 1.00), followed by professional identity and work-family conflict (S(e) = 0.965), and professional identity and occupational calling (S(e) = 0.835). The complete bootstrap results are presented in Supplementary Table 4.

The averaged network, constructed by retaining only stable edges (S(e) > 0.50), revealed that professional identity served as the central hub, directly connecting to occupational calling, job involvement, and work-family conflict. This suggests that while the continuous variable network showed good stability, the connections involving categorical variables (education, professional title) were less robust and should be interpreted with caution. Based on this stable core structure, we further examined the conditional probabilities of low occupational calling given different parent node states (Table 4).

### Probability inference for the current status of occupational calling among psychiatric nurses

Bayesian network models can infer the probability of unknown nodes based on the state of known nodes, thus enabling probability inference for low occupational calling among psychiatric nurses (Table 5). Chi-square tests indicated that combinations of educational level, position, employment type, job involvement, professional identity, and work-family conflict were associated with statistically significant differences in the probability of low occupational calling(*P* < 0.05).

As shown in Table 5, staff nurses with a high level of work-family conflict had a 32.1% probability of low occupational calling, which was significantly higher than that of team leader nurses and head nurses. Nurses with intermediate professional titles had a higher probability of low occupational calling compared with those with primary and senior titles, with the highest probability reaching 34.1%. Additionally, nurses with low job involvement or low professional identity also exhibited higher probabilities of low occupational calling, with the peak probabilities reaching 26.7% and 31.8%, respectively. These findings suggest that nursing managers should consider targeted strategies based on these correlated factors associated with low occupational calling among psychiatric nurses.

**Table 4:** Bootstrap edge strengths for the Bayesian network.

From	To	S(e)	Direction	Stability	From	To	S(e)	Direction	Stability
Job involvement	Professional identity	1	0.405	Very high	Occupational calling	Work-family conflict	0.135	0.519	Low
Professional identity	Job involvement	1	0.595	Very high	Education	Work-family conflict	0.125	0.48	Low
Professional identity	Work-family conflict	0.965	0.933	Very high	Professional title	Work-family conflict	0.125	0.52	Low
Work-family conflict	Professional identity	0.965	0.067	Very high	Education	Occupational calling	0.115	0.522	Low
Occupational calling	Professional identity	0.835	0.281	High	Job involvement	Education	0.095	0.474	Very low
Professional identity	Occupational calling	0.835	0.719	High	Work-family conflict	Education	0.095	0.526	Very low
Job involvement	Work-family conflict	0.285	0.526	Low	Occupational calling	Education	0.085	0.471	Very low
Work-family conflict	Job involvement	0.285	0.474	Low	Education	Job involvement	0.075	0.533	Very low
Occupational calling	Job involvement	0.245	0.51	Low	Professional title	Job involvement	0.075	0.467	Very low
Education	Professional identity	0.185	0.486	Low	Education	Professional title	0.065	0.492	Very low
Professional title	Professional identity	0.185	0.514	Low	Job involvement	Professional title	0.055	0.545	Very low
Education	Job involvement	0.165	0.515	Low	Work-family conflict	Professional title	0.055	0.455	Very low
Job involvement	Occupational calling	0.145	0.552	Low	Occupational calling	Professional title	0.045	0.511	Very low
Work-family conflict	Occupational calling	0.145	0.448	Low	Education	Occupational calling	0.035	0.486	Very low
					Professional title	Occupational calling	0.035	0.514	Very low
					Education	Work-family conflict	0.025	0.48	Very low

S(e) represents the frequency of each directed edge appearing across 200 bootstrap samples. Direction indicates the proportion of times the edge appeared in the specified direction when the edge was present (values close to 0.5 suggest bidirectional uncertainty; values close to 1.0 suggest strong directional consistency). Edges with S(e) > 0.50 are considered stable.

## Discussion

### Key correlated factors of occupational calling in psychiatric nurses by LASSO regression and Bayesian network models

We found that education, position, employment type, professional title, job involvement, professional identity, and work-family conflict were significantly correlated with occupational calling among psychiatric nurses, which is consistent with the findings of previous studies^3,11–14^. To further explore the interrelationships among these correlated factors, a Bayesian network model was constructed, revealing complex network connections between factors associated with low occupational calling in psychiatric nurses. Specifically, position, professional title, job involvement, professional identity, and work-family conflict were directly linked to occupational calling. In contrast, employment type and education showed indirect associations with occupational calling through their relationships with professional identity and job involvement.

Bootstrap stability analysis provided empirical support for the robustness of the core network structure composed of professional identity, occupational calling, job involvement, and work-family conflict. Professional identity emerged as the most stable node, maintaining connections with all other continuous variables across bootstrap samples. This finding further supports the important role of professional identity in psychiatric nurses’ occupational calling^14,15^. However, the connections involving categorical variables (education, professional title, position, employment type) showed lower stability (S(e) <0.50) in bootstrap resampling, suggesting that these relationships may be more sensitive to sample variation. This methodological insight complements network interpretation: although these demographic factors were selected by LASSO and included in the network structure, their associations should be interpreted with caution. This observation indicates the need for larger and more diverse samples to support the important role of occupational calling among nurses^16^.

**Table 5:** The conditional probabilities of low occupational calling among psychiatric nurses.

Variables		Work-family conflict	Probability of low occupational calling(%)	Variables		Work-family conflict	Probability of low occupational calling(%)
Education	Bachelor’s degree	Low	24.5	Job involvement	Low	Low	24.8
		Medium	25.9			Medium	24.7
		High	29.1			High	26.7
	Associate degree	Low	21.3		Medium	Low	14
		Medium	23.8			Medium	16.4
		High	23.3			High	15.5
POST	Nurse	Low	26.2		High	Low	8.4
		Medium	28.9			Medium	6.5
		High	32.1			High	4.1
	Nursing Team leader	Low	12.7	Professional identity	Low	Low	30.2
		Medium	8			Medium	30.6
		High	3.1			High	31.8
	Head nurse	Low	0.6		Medium	Low	15.3
		Medium	0.5			Medium	15.4
		High	0.3			High	14.6
Employment type	Establishment staff	Low	8.8		High	Low	2.9
		Medium	15.3			Medium	2.4
		High	18			High	2.1
	Contract based staff	Low	26.3				
		Medium	27.1				
		High	29.1				
Professional title	Junior professional title	Low	34.1				
		Medium	31.4				
		High	29.2				
	Intermediate	Low	20.8				
		Medium	25				
		High	31.3				
	Senior professional title	Low	1.6				
		Medium	7.3				
		High	5.3				

### Factors correlated with occupational calling

#### Educational

The results showed that education was indirectly associated with occupational calling through professional identity, and nurses with higher educational levels were more likely to report low occupational calling. This finding was consistent with the study by Shen et al.^6^ but inconsistent with those of Tan et al. and Yang et al.^17,18^. This discrepancy may be associated with multiple factors: tertiary Grade A hospitals typically have rigorous requirements for nurses, who often undertake heavy clinical workloads alongside numerous non-clinical responsibilities; the current nursing workforce faces a mismatch between the demand for highly educated nurses and the practical challenges they encounter in role transition^19^, leading to the assignment of more challenging tasks to those with advanced academic backgrounds; in addition, the current nursing appraisal system relies mainly on seniority, professional title, and attendance, resulting in a mismatch between work contribution and reward and insufficient recognition of nurses’ work value^6^. These factors are associated with the relatively low professional identity among highly educated psychiatric nurses.

Combined with the finding that nurses with higher educational levels are more likely to report low occupational calling (consistent with Shen et al.^6^ but inconsistent with Tan et al.^17^ and Yang et al.^18^, nursing managers may provide more platforms for highly educated nurses to demonstrate their capabilities and achieve self-worth, thereby fostering an environment that may support professional identity. Furthermore, strengthening training for nurses with lower educational levels to improve their comprehensive literacy may enhance the professional connotation of psychiatric nursing^20^, which in turn is associated with improved professional identity and relatively higher occupational calling.​.

#### Position

Given that staff nurses had a higher probability of low occupational calling (32.1% for those with high work-family conflict) compared with nursing team leaders and head nurses (consistent with Mao et al.^3^ and international evidence on frontline nurses’ workplace stress^21^, nursing managers and team leaders usually exhibit stronger career commitment and achievement motivation. In contrast, front-line nurses may be more susceptible to job burnout due to repetitive daily work and insufficient professional recognition, a pattern that may correlate with reduced occupational calling^22^. Therefore, targeted career guidance, professional development training, and promotion counseling may be provided for front-line nurses to improve their professional identity and occupational calling.

#### Employment type

The results demonstrated that employment type was associated with occupational calling via job involvement. Contract psychiatric nurses had a significantly higher likelihood of low occupational calling than permanent staff, consistent with Peng et al.^23^ and international evidence on employment model impacts on nursing career outcomes^24^. Permanent employees typically access more welfare benefits, training opportunities, and promotion resources; contract nurses often report lower professional value and job involvement, with these factors linked to reduced occupational calling.

In line with the result that contract psychiatric nurses had a significantly higher likelihood of low occupational calling than permanent staff (consistent with Peng et al.^23^ and international evidence on employment model impacts^24^, managers may implement performance-based incentives to enhance contract nurses’ engagement, explore the professional connotation of psychiatric nursing, and improve psychiatric nurses’ social reputation^24^. These interventions may strengthen job involvement among contract psychiatric nurses^25^, supporting their occupational calling.

#### Professional title

We found junior-title nurses had a significantly higher probability of low occupational calling than intermediate and senior-title nurses, consistent with Mao et al.^3^ but inconsistent with Du et al.^26^, and aligned with international evidence linking early-career plateau to reduced work motivation^27^. Junior-title nurses undertake high-risk clinical tasks and experience career development pressure, with greater susceptibility to job burnout^28^ and lower occupational calling, which is associated with professional engagement and well-being^29^.

Considering that junior-title nurses had a significantly higher probability of low occupational calling (up to 34.1%) than intermediate and senior-title nurses (consistent with Mao et al.^3^ but inconsistent with Du et al.^26^, managers may provide targeted training and career support for junior-title nurses to enhance their professional achievement and work value, which may correlate with improved occupational calling.

#### Job involvement

The results showed that job involvement was positively associated with occupational calling, which is consistent with domestic findings of Yang et al.^18^ and international cross-cultural evidence from multi-country nursing research^30^. Existing studies suggest that higher job involvement among nurses is correlated with stronger professional cognition and a deeper perception of self-worth and work value^14^, with positive work-related states also showing associations with improved nursing quality and organizational performance^31^.

Managers should pay attention to the working status of psychiatric nurses. Given the high-pressure and high-risk nature of their work, these nurses are prone to emotional exhaustion. Managers may identify sources of pressure and implement targeted supportive strategies, such as creating a positive and supportive working environment^18^, relieving negative emotions through effective stress relief channels, and establishing an early warning system for aggressive risks to reduce anxiety and fear caused by potential violence. These strategies may be associated with greater job satisfaction and a stronger sense of belonging among psychiatric nurses^32^.

#### Professional identity

Our findings revealed that professional identity is closely associated with occupational calling; among all factors examined, it showed a relatively strong association with occupational calling. Probability inference results indicated that nurses with low professional identity had a probability of up to 31.8% for low occupational calling, a finding consistent with the study by Zhang et al.^15^ and international research on nursing professional identity^33^. This pattern may be closely associated with the high emotional labor inherent in psychiatric nursing, as these nurses need to respond to patients’ distress and confusion while maintaining their own emotional equilibrium. Furthermore, limited opportunities for professional advancement are associated with poorer mental health and work efficiency, a state that may coincide with challenges in professional identity and relatively lower occupational calling^32^.

Based on the positive association between job involvement and occupational calling observed in this study (consistent with Yang et al.^18^ and international cross-cultural evidence^30^, and the finding that nurses with low job involvement had a 26.7% probability of low occupational calling, managers should pay attention to the working status of psychiatric nurses. Given the high-pressure and high-risk nature of their work, these nurses are prone to emotional exhaustion. Managers may identify sources of pressure and implement targeted supportive strategies, such as creating a positive and supportive working environment^18^, relieving negative emotions through effective stress relief channels, and establishing an early warning system for aggressive risks to reduce anxiety and fear caused by potential violence. These strategies may be associated with greater job satisfaction and a stronger sense of belonging among psychiatric nurses^32^.

Therefore, managers are advised to attend to the psychological state and needs of nursing staff, provide learning and development opportunities to facilitate self-improvement and growth. Such support may foster a stronger sense of professional gain and satisfaction, which in turn could be associated with improved professional identity^34^. In addition, recognizing psychiatric nurses through specialized honorary awards may highlight their professional value and cultivate a greater sense of professional mission and honor, which could be positively associated with occupational calling.

Combined with the finding that nurses with low professional identity had a probability of up to 31.8% for low occupational calling (the strongest association among all factors, consistent with Zhang et al.^15^ and international research^33^, managers are advised to attend to the psychological state and needs of nursing staff, provide learning and development opportunities to facilitate self-improvement and growth. Such support may foster a stronger sense of professional gain and satisfaction, which in turn could be associated with improved professional identity^34^. In addition, recognizing psychiatric nurses through specialized honorary awards may highlight their professional value and cultivate a greater sense of professional mission and honor, which could be positively associated with occupational calling.

#### Work-family conflict

The results showed a direct negative association between work-family conflict and occupational calling, contrasting with South African scholars Koekemoer & Olckers^35^ who reported that work-family conflict may indirectly enhance occupational calling via job crafting as a personal resource. In the present study, greater work-family conflict was associated with an increased probability of low occupational calling among psychiatric nurses, potentially reflecting the influence of their specific work characteristics (e.g., high emotional labor, irregular shifts) on the application of such personal coping strategies^16^.​

Given the direct negative association between work-family conflict and occupational calling in the present study, and the increased probability of low occupational calling with greater conflict (contrasting with South African scholars Koekemoer & Olckers^35^ who reported an indirect positive effect via job crafting), supportive workplace measures (e.g., flexible scheduling, work-family balance resources, reasonable workloads) may reduce work-family conflict, which may in turn relate to better mental health and stronger occupational calling among psychiatric nurses.

LASSO analysis revealed robust associations of professional identity and job involvement with occupational calling, consistent with vocational meaning frameworks^14,15^ and international research^34^. Work-family conflict consistently showed adverse associations^16,23,32^, while the contrasting perspective from South African research^35^ highlights the contextual dependence of this relationship, emphasizing the need to integrate structural support and individual coping resources in psychiatric nursing. Demographic variables (e.g., education, professional title) had weaker associations^16^, suggesting psychosocial factors are more core to occupational calling^19^.

## Limitations

Several limitations of this study should be acknowledged. First, the cross-sectional design only captures cross-sectional variable correlations, precluding causal inference and verification of temporal sequence; directed edges in the Bayesian network represent conditional associations rather than causal effects. Second, convenience sampling from a single tertiary Grade A hospital in Mianyang limits generalizability, may introduce selection bias, and the sample may not be representative of psychiatric nurses in other institutions or regions^19^. Third, the modest sample size (*N* = 216) may relate to lower Bayesian network stability; edges for categorical variables (education, professional title, position, employment type) showed low stability (S(e)<0.50) in bootstrap validation and require cautious interpretation. Fourth, only demographic and psychosocial variables were included, while organizational, individual psychological (e.g., resilience) and social factors (e.g., occupational stigma) were not considered, leading to an incomplete occupational calling correlation network^20^. Fifth, sole reliance on self-reported questionnaires may introduce response biases (e.g., social desirability bias), with no objective indicators for cross-validation to ensure measurement accuracy.​.

Future research could adopt longitudinal designs to clarify variable temporal sequences, use multi-center stratified random sampling to expand samples and improve generalizability^19^, include comprehensive variables for a more complete correlation network^20^, and combine questionnaires with objective indicators to reduce measurement bias. Further exploration of the moderating/mediating relationships of key variables (e.g., occupational resilience, organizational support) between psychosocial factors and occupational calling may provide targeted reference for nursing management interventions^27^.​.

## Conclusion

The study showed that psychiatric nurses’ occupational calling is associated with multiple factors, among which professional identity demonstrates a relatively strong and stable association. LASSO coefficient path analysis further confirmed that psychosocial factors (professional identity, job involvement, work-family conflict) were more consistent correlates of occupational calling than demographic characteristics, maintaining non-zero coefficients even under strong regularization. Psychiatric nurses encounter unique challenges related to their specialized constraints and high work risks, which may be associated with their sense of occupational calling.​.

These findings may assist in identifying modifiable factors associated with occupational calling in this population and provide insights for multidimensional management strategies targeting psychosocial factors (professional identity, job involvement, work-family conflict) and key groups (contract nurses, frontline nurses, highly educated nurses). The implementation of these comprehensive strategies may be associated with higher levels of occupational calling among psychiatric nurses, which in turn may correlate with improved overall job satisfaction and enhanced stability of the nursing team.

## Data Availability

The data that support the findings of this study are available within the article. Additional raw data derived from human participants are not publicly available to protect their privacy but are available from the corresponding author on reasonable request, subject to ethical approval.
